# Sarcoma of the Prostate

**Published:** 1918-08

**Authors:** 


**Sarcoma of the Prostate** is reported by B. de Saussine in the Arch. de Med. des Enfants, in a boy 4½ years old. The irritation at first caused frequent voiding of urine but dysuria followed and complete retention requiring the use of the catheter developed in two weeks from the onset of symptoms. Death occurred from ascending infection. Necropsy showed a tumor as large as a mandarin orange, surrounding the prostatic urethra, purulent systitis and some pyelonephritis.

